# Development of Sensor Cells Using NF-κB Pathway Activation for Detection of Nanoparticle-Induced Inflammation

**DOI:** 10.3390/s110707219

**Published:** 2011-07-18

**Authors:** Peng Chen, Satoshi Migita, Koki Kanehira, Shuji Sonezaki, Akiyoshi Taniguchi

**Affiliations:** 1 Cell-Materials Interaction Group, Biomaterials Unit, Nano-Bio Field, National Institute for Materials Science, 1-1, Namiki, Tsukuba, Ibaraki, 305-0044, Japan; E-Mails: chen.peng@nims.go.jp (P.C.); migita.met@tmd.ac.jp (S.M.); 2 Graduate School of Advanced Science and Engineering, Waseda University, 3-4-1 Okubo, Shinjuku, Tokyo 169-8555, Japan; 3 TOTO Ltd. Research Institute, Nakashima 2-1-1, Kokurakita, Kitakyushu, 802-8601, Japan; E-Mails: koki.kanehira@toto.co.jp (K.K.); shuji.sonezaki@toto.co.jp (S.S.)

**Keywords:** sensor cells, TiO_2_ nanoparticles agglomerates, inflammation, NF-κB, Toll-like receptor4

## Abstract

The increasing use of nanomaterials in consumer and industrial products has aroused concerns regarding their fate in biological systems. An effective detection method to evaluate the safety of bio-nanomaterials is therefore very important. Titanium dioxide (TiO_2_), which is manufactured worldwide in large quantities for use in a wide range of applications, including pigment and cosmetic manufacturing, was once thought to be an inert material, but recently, more and more studies have indicated that TiO_2_ nanoparticles (TiO_2_ NPs) can cause inflammation and be harmful to humans by causing lung and brain problems. In order to evaluate the safety of TiO_2_ NPs for the environment and for humans, sensor cells for inflammation detection were developed, and these were transfected with the Toll-like receptor 4 (TLR4) gene and Nuclear Factor Kappa B (NF-κB) reporter gene. NF-κB as a primary cause of inflammation has received a lot of attention, and it can be activated by a wide variety of external stimuli. Our data show that TiO_2_ NPs-induced inflammation can be detected by our sensor cells through NF-κB pathway activation. This may lead to our sensor cells being used for bio-nanomaterial safety evaluation.

## 1. Introduction

Nanotechnology is a highly active field. A large variety of nanomaterials have be prepared and investigated for numerous potential technological applications. For example, many classes of metallic, metallic oxide, organic, or organic-inorganic biocompatible nanoparticles have been widely synthesized and commercialized. Given their unique properties, there are high hopes for these nanoparticles to be employed for biomedical applications, for instance as drug-delivery agents [1,2] or imaging contrast agents [3,4], where the nanoparticles would be directly ingested or injected into the body. It is forecasted that the production of nanoparticles will increase to 58,000 tons per year from 2011 to 2020 [5]. Although these nanomaterials, such as nanotubes, nanofibers, fullerene derivatives, quantum dots, are expected to be widely applied, little is known about their effects on the environment and their health effects upon human exposure.

A controversy [6–16] erupted after an investigation revealed that TiO_2_ NPs found in sunscreens could cause brain damage in mice [17]. Of note, the “nanotoxicity” of TiO_2_ NPs is dependent on their particle size. TiO_2_ NPs are usually recognized as having three morphological states: primary particles, aggregates, and agglomerates. Primary particles are single crystals bound by crystal planes. Aggregates are sintered primary particles connected by crystal faces. TiO_2_ NPs are multiple primary particles and/or aggregates held together by Van der Waal’s forces. In general, for commercial products such as pigments, primary particle sizes within 0.2–0.3 μm in diameter are used, while almost no particles less than 0.1 μm can be found. It is thus considered practical to examine particles with sizes ranging from 200 nm to less 1 μm in diameter [18]. In our previous study, based on the mRNA expression of biomarkers of stress, inflammation, and cytotoxicity in TiO_2_ NPs-exposed cells, we found that compared with small TiO_2_ NPs (about 170 nm in average diameter), large TiO_2_ NPs (about 600 nm in average diameter) have a larger effect on cell viability and gene expression, such as for the expression of inflammation markers interleukin-6 (IL-6), interleukin-8 (IL-8), tumor necrosis factor (TNF), and the universal toxicity marker heat shock protein [19]. This is consistent with a result reported by the IARC (1989) showing that high doses of TiO_2_ can cause inflammation (lung damage or fibrosis) *in vivo*.

Inflammation is the earliest response to outside infection or damage to the body, and can arouse an immune response against invading pathogens like bacteria and viruses. In addition, a number of chronic diseases cause inflammation at the incunabular stage, such as heart disease, diabetes and gout. However, at the same time, pre-inflammation can be detrimental to the body, especially in chronic inflammation that can lead to rheumatoid arthritis, asthma, or cancer. Accordingly, in order to prevent disease, it is necessary to detect unwanted pre-inflammation activation in a timely manner.

NF-κB as a primary cause of inflammation has received a lot of attention. As NF-κB complexes can be found in all cell types, NF-kappa protein is regarded to be an adjustor of the immune response, inflammatory processes, and proliferative and apoptotic regulation of the cell. Furthermore, many different stimuli associated with stress or injury are related with NF-κB, such as cytokines (e.g., IL-1, IL-6, IL-8, TNF), bacterial and viral products (e.g., lipopolysaccharide (LPS), sphingomyelinase, double-stranded RNA), proapoptotic and necrotic stimulus (e.g., oxygen free radicals, ultraviolet light) [20]. Therefore, NF-κB is a key element for pre-inflammation detection. This has led to great interest in investigating the inducing mechanisms of NF-κB. A simplified model would be as follows. Inflammatory signals start from the receptors, such as TLR4, which can form a complex with a variety of inducements on the surface of the cell membrane, and result in an inflammatory signal sent out to the cytoplasmic side of the membrane. Then the inflammatory signal stimulates the activation of NF-κB in the cytosol. When NF-κB is activated, it dissociates from its inhibitory protein IκB. Then NF-κB is released and is allowed to migrate to the nucleus of the cell where it activates the transcription of pre-inflammatory genes.

Live cell-based biosensors have received increasing attention due to their high sensitivity and accuracy. Live cell biosensors have great advantages in both detection limitation and testing speed compared with traditional testing methods, such as cell viability tests [21]. In our laboratory, using molecular biology techniques, we have created next-generation sensor cells that can detect changes in gene expression in response to drugs and other external stimuli. Heat shock protein 70B’ (HSP70B’) gene-containing sensor cells can represent a protein related cytotoxic response caused by cadmium chloride and other cellular toxins [22,23]. Another sensor cell system with an introduced B-cell translocation gene 2 (BTG2) promoter and a reporter gene (luciferase or red fluorescence protein) can identify DNA damage-related cytotoxic stimulation [21,24].

Considering that the use of nanomaterials and public exposure is increasing, accurate and fast measurement techniques to evaluate their safety are required. In this work, sensor cells for detecting pre-inflammation through the NF-κB pathway were constructed. We tested the feasibility of detecting inflammation with our sensor cells by using the strong inflammation stimulator, LPS. Then we tested the exposure of TiO_2_ NPs to the sensor cells. This system is a potentially sensitive and convenient method for verifying the safety of nanomaterials.

## 2. Experimental Section

### 2.1. Preparation of TiO_2_ NPs

Two TiO_2_ NPs samples were used with two different sizes, which were described in a previous study [19]. TiO_2_ NPs (TiO_2_: Degussa Aeroxide P25) were dispersed in distilled water and autoclaved at 120 °C for 20 min. The TiO_2_ NPs suspension was sonicated for 10 min at 200 kHz by a high frequency ultrasonic sonicator (MidSonic 600, Kaijo, Japan) after cooling to room temperature. This TiO_2_ NPs suspension was named “large TiO_2_ NPs”. Simultaneously, a fraction of the TiO_2_ NPs was selected by centrifugation (700× g, 5 min) at 4 °C. The supernatant was carefully recovered and named “small TiO_2_ NPs”. The concentrations of both TiO_2_ NPs samples were determined using a UV-VIS spectrophotometer (UV-1600, Shimadzu, Japan). Both samples were adjusted to the same concentration by adding distilled water, and then stored at 4 °C until used. The particle size distribution was measured by dynamic light scattering (ZetasizerNano-ZS, Malvern Instruments, UK), according to the manufacturer's instructions. Both samples were diluted by cell culture medium supplemented with 10% fetal bovine serum, and the particle sizes of small and large TiO_2_ NPs samples were determined to be 169 ± 55 nm and 572 ± 243 nm, respectively. After incubation at 37 °C for 24 h, the size distribution of both particle samples remained mostly unchanged. The agglomerate TiO_2_ NPs sizes were stable for at least 6 months.

### 2.2. Cell Cultures

The mouse embryonic fibroblast cell line NIH/3T3 was cultured in Dulbecco’s modified Eagle’s medium (Sigma) supplemented with 10% fetal bovine serum (Biowest), 100 U/mL penicillin, and 100 μg/mL streptomycin (Invitrogen) at 37 °C in a humidified atmosphere with 5% CO_2_.

### 2.3. Preparation of Sensor Cells

Two reporter gene plasmids, pGL3-Control vector (pGL3 plasmid) (Promega) and pGL4.32[luc2P/NF-κB-RE/Hygro] vector (NF-κB reporter plasmid) (Promega), were employed as a blank control and an NF-κB signal reporter, respectively. Both contain SV40 promoter and enhancer sequences, resulting in strong expression of luc in many types of mammalian cells. But compared with the blank control, the NF-κB reporter plasmid can promote luc expression when NF-κB is released by a stimulator. The pRL-CMV vector, which contains the CMV promoter upstream of the *Renilla* luciferase gene (Promega), was transfected to serve as an internal control for variations in transfection efficiency. The TLR4 expression vector, pUNO1-hTLR04a (InvivoGene), was also introduced into the sensor cells to improve the detection ability. All transfection experiments were performed with Lipofectamine^™^ LTX Reagent (Invitrogen) according to the supplier’s protocol. NIH/3T3 cells were seeded in 24-well plates. After overnight incubation, cells were co-transfected with a reporter gene plasmid (pGL3 plasmid or NF-κB reporter plasmid) and pRL-CMV vector plasmid using Lipofectamine^™^ LTX Reagent (Invitrogen), and medium was renewed after 4∼6 h post-transfection.

### 2.4. Inflammation Stimulus Exposure (LPS or TiO_2_ NPs)

LPS *E. coli* J5 was purchased from Calbiochem (EMD Biosciences, Inc., San Diego, CA, USA). LPS or two kinds of TiO_2_ NPs (large TiO_2_ NPs and small TiO_2_ NPs) were added as stimuli to culture medium immediately before the medium was applied to the cells. One day after transfection, the culture medium was replaced by medium containing the stimuli at the intended concentration, and then the cells were harvested after the indicated times.

### 2.5. Detection of Inflammation Caused by Stimuli Using the Sensor Cells

Luciferase activity was assessed by the Dual-Luciferase Reporter Assay System (Promega) as described in our previous work [24]. The induction ratio of the stimuli (LPS or TiO_2_ NPs) response was evaluated as follows. The cells were transfected with pGL3 plasmid or NF-κB reporter plasmid as described above. With or without stimulus exposure, the luciferase induction ratio was evaluated with the luciferase activity measured. The stimulus response was calculated by the luciferase induction ratio of the sample with exposure to the stimulus divided by that without exposure.

All results from at least three independent tests were evaluated using the Dunnet multiple comparison test. Results are expressed as means ± standard deviation (S.D.).

## 3. Results and Discussion

### 3.1. Experimental Results

**Figure 1.**
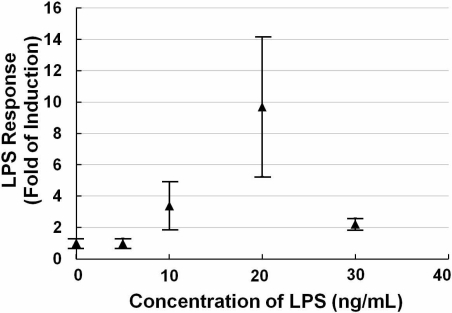
LPS response of NIH/3T3 cells transfected with TLR4 expression vector and NF-κB reporter plasmid. Scattergram of the LPS response (fold induction) of NIH/3T3 cells transfected with TLR4 expression vector and NF-κB reporter plasmid exposed to different concentrations (0, 5, 10, 20, and 30 ng/mL) of LPS for 12 h. Each point was produced from at least 3 independent measurements. All values are presented as means ± S.D. (n ≥ 3).

**Figure 2.**
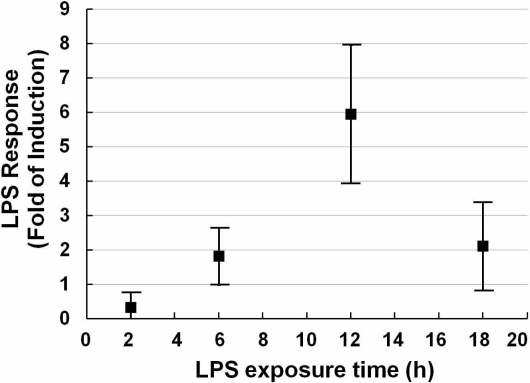
LPS exposure time-course of NIH/3T3 cells transfected with TLR4 expression vector and NF-κB reporter plasmid. Scattergram of LPS response (fold induction) of NIH/3T3 cells transfected with the TLR4 expression vector and NF-κB reporter plasmid exposed to 20 ng/mL LPS with a series time of exposure. Each point was produced from at least 3 independent measurements. All values are presented as means ± S.D. (n ≥ 3).

In order to ascertain the feasibility of detecting inflammation by our sensor cells, a series of experiments were conducted, such as to determine the dose-response (Figure 1) and exposure time-course of LPS (Figure 2). The LPS response data were calculated by the intensities of relative luciferase activities as described in the experimental section.

Figure 1 shows a scattergram of the LPS response of NIH/3T3 cells transfected with the TLR4 expression vector and NF-κB reporter plasmid exposed to different concentrations (0, 5, 10, 20, and 30 ng/mL) of LPS for 12 h. The data show that at the low concentration, the LPS response did not change much. And with increasing concentrations of LPS, the LPS response increased sharply. After it reached a peak (20 ng/mL), the LPS response decreased due to the death of the cells. We observed that around a concentration of 20 ng/mL LPS, a small quantity of cells was suspended in the medium, indicating cell death. Higher concentrations (50 ng/mL and 100 ng/mL LPS) were also tested, and a large number of cells died resulting in a low LPS response (a little higher than the negative control, data not shown). Therefore, we fixed the concentration at 20 ng/mL LPS for follow-up testing. This data also suggested that the detection range of our sensor cells would be from 10 ng/mL to 20 ng/mL for LPS.

After fixing the concentration of the stimulus, the exposure time-course was studied. A series of exposure times (2, 6, 12, 18 h) was investigated. As shown in Figure 2, with the extension of exposure time, the relative luciferase activity was increased until 12 h after which it decreased. Although there is a lot of variability compared with its neighbors, it shows a preferable LPS response.

**Figure 3.**
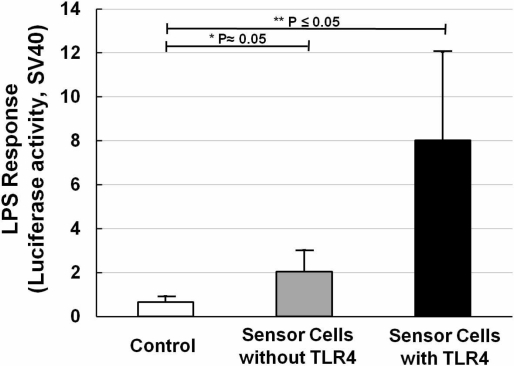
Effect of TLR4 expression vector on LPS response. Histogram of the LPS response (luciferase activity, SV40) of control cells (with TLR4 expression vector and NF-κB reporter gene, but without LPS exposure, open bar) and relative luciferase activities (SV40) of NIH/3T3 sensor cells without (solid gray bar) and with (solid black bar) TLR4 exposed to 20 ng/mL LPS for 12 h. All values are presented as means ± S.D. (n ≥ 3).

NIH/3T3 cells transfected with NF-κB reporter plasmid were designated as sensor cells for inflammation. In the interest of clarity, Figure 3 shows the LPS response of sensor cells without LPS stimulation (Figure 3, open bar), and also sensor cells without (Figure 3, solid gray bar) or with (Figure 3, solid black bar) TLR4 expression vector transfection, for which LPS was used at a concentration of 20 ng/mL for 12 h. It is clear that with only the NF-κB reporter gene, a two-fold increase in the LPS response can be observed. After adding the TLR4 expression vector, the LPS response significantly increased by about 4-fold compared with that without the TLR4 expression vector. And compared with the control sample (without LPS stimulation), it was about 8-times higher. The P values were calculated and are shown. Furthermore, NF-κB responding sensor cells, K562 [25], were also tested to compare them under the same conditions. The resulting data (not shown) showed that our sensor cells with the TLR4 expression vector have an even higher LPS response than the K562 cells. The data thus confirmed that our sensor cells with the NF-κB reporter gene and TLR4 expression vector are highly capable of detecting inflammation through the NF-κB pathway caused by LPS.

**Figure 4.**
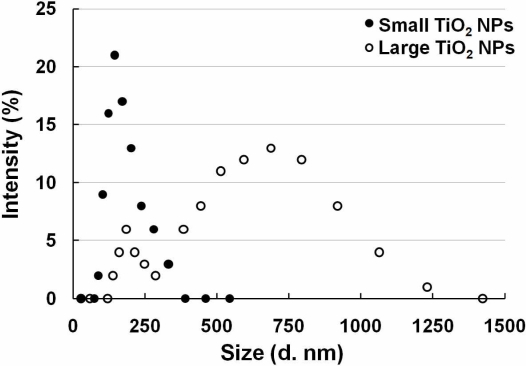
TiO_2_ NP agglomerate size distributions measured by dynamic light scattering analysis. Open diamonds show the size distribution of small TiO_2_ NPs, and black solid diamonds show the size distribution of large TiO_2_ NPs.

Since our sensor cells showed promise to detect inflammation through NF-κB pathway activation caused by LPS, we used them to evaluate the safety of nanomaterials. Two different sizes of TiO_2_ NPs agglomerates were prepared from the same primary TiO_2_ NPs (P25) and sensor cells were exposed to them. The two sizes of TiO_2_ NPs were measured and are abbreviated as small TiO_2_ NPs (169 ± 55 nm) and large TiO_2_ NPs (572 ± 243) (Figure 4).

**Figure 5.**
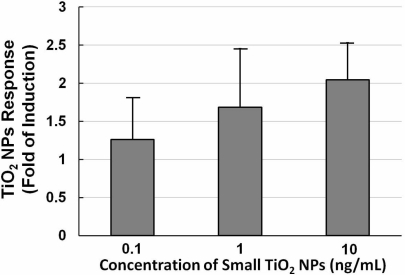
NP dose-response of NIH/3T3 cells transfected with TLR4 expression vector and NF-κB reporter plasmid. Histogram of TiO_2_ NPs response (fold induction) of NIH/3T3 cells transfected with the TLR4 expression vector and NF-κB reporter plasmid exposed to different concentrations (0.1, 1, and 10 ng/mL) of small aggregated TiO_2_ NPs for 10 h. Each point was produced from at least three independent measurements. All values are presented as means ± S.D. (n ≥ 3).

Figure 5 shows a histogram of the NPs dose-response of NIH/3T3 cells transfected with the TLR4 expression vector and NF-κB reporter plasmid exposed to different concentrations (0.1, 1, and 10 ng/mL) of small TiO_2_ NPs for 10 h. The data show that the TiO_2_ NPs dose-response was not large. With increasing concentrations of TiO_2_ NPs, the TiO_2_ NPs response increased slightly, so we therefore set the concentration at 10 ng/mL for subsequent experiments.

**Figure 6.**
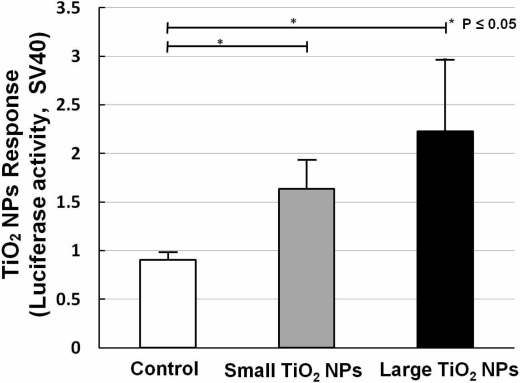
Large and small TiO_2_ NPs response of NIH/3T3 cells transfected with TLR4 expression vector and NF-7κB reporter plasmid. Histogram of TiO_2_ NPs response (luciferase activity, SV40) by prepared sensor cells without (open bar) and with exposure to 10 ng/mL small aggregated TiO_2_ NPs (solid gray bar) and large aggregated TiO_2_ NPs (solid black bar) for 10 h. Each plot was produced from at least three independent measurements. All values are presented as means ± S.D. (n ≥ 3).

Two kinds of TiO_2_ NPs were used, similar to what we previously used for mRNA expression analysis [19]. In our former work, we measured the mRNA expression of biomarkers related to stress, inflammation, and cytotoxicity in TiO_2_-NPs-exposed cells, and found that compared with the small aggregated TiO_2_ NPs (about 170 nm in average diameter), large aggregated TiO_2_ NPs (about 600 nm in average diameter) showed a larger effect on cell viability and gene expression, such as for inflammation markers IL-6, IL-8, and TNF [19]. A bar chart (Figure 6) shows TiO_2_ NPs response data collected by sensor cells without (open bar) and with exposure to 10 ng/mL small aggregated TiO_2_ NPs (solid gray bar) and large aggregated TiO_2_ NPs (solid black bar) for 10 h. Compared with the control sample (without TiO_2_ NPs stimulation), the sensor cells showed a clear TiO_2_ NPs response for both sizes. The P values also affirm this response. But although we introduced the TLR4 expression vector, the response intensity level was not increased to the same extent as with LPS.

### 3.2. Discussion

In our previous work [19], we measured the mRNA expression of several notable biomarkers. Some biomarkers, such as IL-6, IL-8, and TNF, showed a response upon exposure to TiO_2_ NPs. These biomarkers are closely tied into NF-κB pathway activation. But because of the complex cellular environment, and the stability of mRNA, a response based on the luciferase activity of sensor cells using only the transfection of an NF-κB reporter gene was unsatisfactory. So we first tried to improve the ability of sensor cells to detect pre-inflammation through the NF-κB pathway.

TLR4, a transmembrane protein, plays a very important role in the innate immune system by recognizing LPS and triggering an immune response through the NF-κB pathway. It is well known that members of the TLR family can be found on the surfaces of many mammalian cells, and humans have at least ten TLRs, several of which have been shown to play important roles in innate immune recognition. Although different human TLRs are activated in response to different ligands, many of them use NF-κB pathway activation to promote gene expression to initiate an inflammatory response. TLRs are abundant on the surfaces of macrophages and neutrophils, as well as on epithelial cells lining the lung and gut. They act as an alarm system to alert both the innate and adaptive immune systems that an infection is brewing. In order to greatly improve the performance of our sensor cells for detecting pre-inflammation through NF-κB pathway activation, a TLR4 expression vector was transfected into NIH/3T3 cells. The pGL3 plasmid was also used as a negative control in parallel trials.

Additionally, LPS, a major component of the outer membrane of Gram-negative bacteria, has been widely used for experimental research due to its role in activating many transcription factors. LPS forms a complex with LPS-binding protein (LBP) in the blood, and the complex binds to the GPI-anchored receptor Cluster of differentiation 14 (CD14) on the surface of cell membranes. Then it forms a complex with TLR4and MD2 (Lymphocyte antigen 96). Through this process, TLR4 becomes activated, which result in a series of responses on the cytoplasmic side of the membrane, including interaction with myeloid differentiation protein 88 (MyD88), activation of interleukin-1 receptor associated kinase (IRAK), association with tumor-necrosis-factor-receptor-associated factor 6 (TRAF6), and finally degradation of IκB. Eventually, NF-κB promotes LPS immune and inflammatory responses in the nucleus as described previously [26].

We did a series of TiO_2_ NPs tests as we did for LPS, including exposure time-course and dose-response investigation. Exposure time-course data indicated that the response peaked at 10 h and declined thereafter for TiO_2_ NPs. Concerning the inconsistency with the LPS data, one reason might be that the mechanism by which TiO_2_ NPs induces activation of NF-kB is different from that of LPS. Another speculative possibility is the inhibitory effect of the TLR4 expression vector. It has been reported that, after an initial inflammatory stage, TLRs are down regulated in response to titanium particles, possibly to inhibit excessive inflammation [27]. This leads us to surmise that not only is TLR4 involved in the nanoparticle-induced immune reaction, but that NF-κB activation is promoted by two or more receptors from the TLR family or from other families, in addition to other possible causes.

The dose-response data for TiO_2_ NPs showed that the dose did not have a large effect. We have already shown that NF-κB responsive genes, such as IL-6 mRNA, increased about 10-fold by TiO_2_ NPs treatment [19]. In our opinion, these small differences were due to the low sensitivity of the testing method. Although the response level was not high, our sensor cells showed a TiO_2_ NPs response, and can possibility be used to assess the inflammation started by TiO_2_ NPs. In our opinion, the immune response induced by TiO_2_ NPs is a complex process in which several receptors might contribute to inflammation activation. In future work, we will try to incorporate multiple concurrent receptors to detect nanomaterials.

## 4. Conclusions

In this study, in order to create an effective detection system for inflammation by monitoring of NF-κB pathway activation, a TLR4 expression vector and NF-κB reporter plasmid were transfected into NIH/3T3 cells to construct a live cell-based biosensor. The LPS response of our sensor cells detected inflammation through NF-κB pathway activation (around an 8-fold increase), and the data showed a significant dose-effect in a time-dependent manner. For nanoparticles (TiO_2_ NPs), a TiO_2_ NPs response by the sensor cells was found; however, the detectability requires improvement. Difficulties in the detection could be caused by confounding factors such as concurrent receptors. By combining this system with other methods, our sensor cells could potentially be used to evaluate the safety of bio-nanomaterial.
